# Paracetamol Removal from Aqueous Media Through Fenton Reaction Using ZSM-5 Zeolite Produced from Fly Ash

**DOI:** 10.3390/molecules31071104

**Published:** 2026-03-27

**Authors:** Nuno Horta, Sofia Martins, Hugo F. Silva, Nelson Nunes, Ana S. Mestre, Ana P. Carvalho, Angela Martins

**Affiliations:** 1 Departamento de Engenharia Química, Instituto Superior de Engenharia de Lisboa, Instituto Politécnico de Lisboa, R. Conselheiro Emídio Navarro, 1, 1959-007 Lisboa, Portugal; 2 Centro de Química Estrutural, Faculdade de Ciências, Institute of Molecular Sciences, Universidade de Lisboa, Campo Grande, 1749-016 Lisboa, Portugalana.carvalho@ciencias.ulisboa.pt (A.P.C.); 3 Departamento de Química e Bioquímica, Faculdade de Ciências, Universidade de Lisboa, Ed.C8, Campo Grande, 1749-016 Lisboa, Portugal

**Keywords:** fly ash, ZSM-5 zeolite, paracetamol, Fenton reaction, kinetic parameters

## Abstract

The purpose of this study is the exploration of the catalytic performance of a ZSM-5 zeolite produced from iron-rich fly ash, without any additional iron loading, in removing paracetamol via a heterogenous Fenton reaction. The structural and textural characterization by powder X-ray diffraction and N_2_ adsorption isotherms showed that a pure ZSM-5 phase was synthesized, but lower crystallinity and textural parameters were obtained when compared with commercial ZSM-5. The XPS analysis revealed significant amounts of iron and yttrium, which enhanced the electronic properties of the samples’ surface when compared with iron-impregnated commercial ZSM-5. The catalytic reaction was followed through UV-spectroscopy and kinetic models were applied to the data; the best fit was obtained for a pseudo-first-order model. All fly ash-based zeolites showed increased paracetamol removal when compared with commercial iron-loaded ZSM-5, which may be attributed to the more disordered structure, able to accommodate large paracetamol species (dimers). On the other hand, the effect of yttrium on the electronic properties of iron sites may increase the ^●^OH radical formation, thus increasing the paracetamol removal rate, despite the progressive drop on paracetamol removal upon regeneration–reuse cycles due to Fe leaching.

## 1. Introduction

The supply of drinking water is one of the greatest concerns for industrialized societies. Among persistent organic pollutants (POPs), pharmaceuticals are of particular concern due to their increasing consumption and unclear cumulative effects on human health and the environment [1,2]. Paracetamol (PA), also designated as acetaminophen, is a drug used to treat fever and mild to moderate pain. PA toxicity was evaluated in hosts like bacteria, algae, protozoan, and fishes, showing that long-term exposure can cause genetic damage, oxidative lipid degradation, and denaturation of cellular proteins [3,4]. On the other hand, by-products generated during PA degradation, such as hydroquinone, benzoquinone or nitrophenols [5,6] can be as harmful or even more than the original compound [3]. Thus, the removal of PA and/or its degradation products from wastewater is mandatory to avoid harmful health effects on living beings and environment. Several studies point out that even after conventional wastewater treatment processes, a significant amount of PA may remain in the environment either in its original form or as by-products; therefore, more efficient processes are required to remove or convert it into innocuous compounds [7]. In recent decades, several studies have shown that advanced oxidation processes (AOPs) are effective for degrading organic molecules, including pharmaceutical compounds. AOPs generate highly reactive and non-selective oxidant species, such as hydroxyl radicals, ^●^OH, which can transform organic molecules into simple H_2_O and CO_2_, as final products [2,3]. A typical homogeneous Fenton reaction follows a free radical mechanism, involving the interaction between Fe^2+^ and H_2_O_2_ in acidic conditions. The ^●^OH radical species are generated, promoting the degradation of a wide range of organic molecules, Equations (1)–(3).

Fe^2+^ + H_2_O_2_ → Fe^3+^ + OH^−^ + ^●^OH (1)

Fe^3+^ + H_2_O_2_ → ^●^OOH + Fe^2+^ + H^+^ (2)

RH + ^●^OH → H_2_O + ^●^R → Degradation → Mineralization (3)

Recent computational studies have complemented experimental investigations by elucidating molecular-level degradation pathways. For instance, Mai et al. [8] employed density functional theory (DFT) calculations to investigate the initial degradation mechanism of salicylic acid via electrochemical oxidation, providing insights into intermediate product formation and reaction energetics. Such computational approaches are increasingly valuable for understanding complex advanced oxidation process mechanisms that are difficult to characterize experimentally.

The main drawback of the homogeneous Fenton reaction is the need to operate in a very acidic environment, typically at a pH between 2 and 4, to avoid the precipitation of inactive iron oxyhydroxides species [9], which form iron sludges. The costs associated with the removal of these sludges, effluent treatment and catalyst loss, make the quest for alternative heterogeneous catalysts most appealing. Several heterogeneous iron-based catalysts have been studied, which allow operation in a wider pH range with reuse possibility [10,11]. Fe-containing zeolites are among the most interesting candidates to replace homogeneous catalysts, due to their crystalline, ordered porous structure, and ability to anchor metal species both at the external and inner surface. Thus, hetero-Fenton reactions occur, according to Equation (4), where Z-Fe^2+^ and Z-Fe^3+^ are the iron species supported on the zeolite [12].

Z-Fe^2+^ + H_2_O_2_ → Z-Fe^3+^ + OH^−^ + ^●^OH (4)

Several studies report the use of iron-loaded zeolites as Fenton and photo-Fenton catalysts for the removal of a wide range of organic pollutants [12,13,14,15]. The catalytic performance of these materials strongly depends on the type of zeolite structure and the Si/Al ratio [12]. On the other hand, high iron loadings may result on important losses of microporous volume and clogging of pore openings, which hinders access to the inner porosity [7]. Iron-loaded ZSM-5 (MFI structure), one of the most used zeolites as a heterogeneous catalyst or catalyst support, has stood out as an effective Fenton or photo-Fenton catalyst [7,15]. Recently, modified Fe-ZSM-5 was studied in the removal of methylene blue dye [14]. In this case, prior to iron loading, commercial ZSM-5 was modified through desilication, followed, in some cases, by acid leaching. The generated mesoporosity facilitated diffusion, leading to an improved catalytic performance, but also increased the catalyst preparation costs. Accordingly, low cost and effective synthesis procedures for iron-loaded zeolites are desired.

Fly ash (FA) is a by-product of coal combustion in thermal power plants, with a worldwide production of about 780 million tonnes, but a low recycling rate of around 25%, mostly as an additive for cement (≈20%) [16,17]. Therefore, the disposal of large amounts of this industrial solid waste has become a serious environmental concern and, from the perspective of Circular Economy, is still a resource to be explored. The chemical composition of FA depends on the coal origin and technological processes used in thermal power plants. According to Vassilev and Vassileva [18], about 188 mineral groups have been identified in FAs. The chemical composition of FA, in order of decreasing amounts, includes oxygen, silicon, aluminum, calcium, iron, carbon, potassium, magnesium, and sulfur. Rare earth elements (REEs) have also been detected. The presence of these elements has been highlighted given the scarcity and critical need for various industry sectors like automotive, electronics, energy, etc., enhancing the feasibility of extracting REEs from fly ashes [19,20].

Since all fly ash materials are rich in mineral phases containing Si and Al—the main components of zeolites—the process of turning FA into zeolites has attracted growing interest, as it reduces waste disposal and converts the material into a valuable product. The synthesis of several zeolite structures has been reported [21,22], including ZSM-5 [23].

Zeolite synthesis from FA requires digestion in alkaline media to dissolve the insoluble glass phase and crystalline phases such as mullite and quartz, thereby transforming the building blocks into the aluminosilicate framework typical of zeolites [21,24]. However, this process raises concerns since it could mobilize toxic heavy metals, contaminating the environment. Feng et al. [21] investigated this issue and concluded that less than 20% of heavy metals (copper, chromium, and lead) from FA entered wastewater, whereas the rest, along with almost all cadmium, iron, and nickel, was incorporated into the synthesized zeolite structures. Moreover, these elements remained in the zeolite structure without further leaching, regardless of the pH conditions; thus, the synthesized zeolites are safe for applications without environmental contamination.

The application of FA-based zeolites has been reviewed in several case studies [16,22,25,26]. Most examples refer to their use as adsorbents, including the removal of heavy metals [27], dioxins [28] and CO_2_ [29], among others. Although catalytic applications are less explored, a few examples include fast pyrolysis of biomass [30] and conversion of glucose to 5-hydroxymethylfufural (HMF) [31]. Within the scope of Fenton catalysis, a recent study reported tetracycline degradation in Fenton-like oxidation reactions using Fe-, Cu- and Fe/Cu-loaded ZSM5-5 zeolite produced from fly ash and rice husk [32]. In the same context, the present study deals with the synthesis of ZSM-5 zeolite from FA. Given the rich composition of iron and other elements in the raw fly ash used, the potentialities of FA-ZSM5 as Fenton catalysts are explored without further addition of metal ions. Thus, the major goal of this work is to valorize industrial waste, eliminating additional steps and costs associated with metal ion introduction, to prepare an effective Fenton catalyst for paracetamol removal from aqueous media.

## 2. Results

### 2.1. Materials Characterization

The characterization of the FA sample was performed in a previous study [33], where the bulk mineral content was examined, as well as the leaching effect when FA contacted with aqueous media. In brief, proximate analysis of the FA revealed low moisture and volatile matter contents, with almost 95 wt.% ash. The material’s crystalline structure was characterized by XRD diffractometry, showing two predominant phases: quartz (~77.9%), mullite (~8.0%), and small amounts of magnetite. BET surface area analysis yielded a specific surface area of 13.2 m^2^ g^−1^, and particle size distribution ranged from 0.5 to 10 μm.

Inductively coupled plasma atomic emission spectrometry (ICP-AES) analysis revealed that the major components of fly ash were aluminum at 10.1 wt% (3.7 mmol g^−1^) and silicon at 26.5 wt% (9.5 mmol g^−1^), with iron present at approximately 4.3 wt% (0.8 mmol g^−1^). Additional minor constituents comprised magnesium, potassium, calcium, sodium, manganese, zinc, and other trace elements.

The aqueous leachate data showed that Ca, Mg, and K were released most significantly, whereas Al and Si were released to the environment at less than 1%. Accordingly, FA can be used safely without significant release of harmful elements through leaching [33].

**Figure 1:**
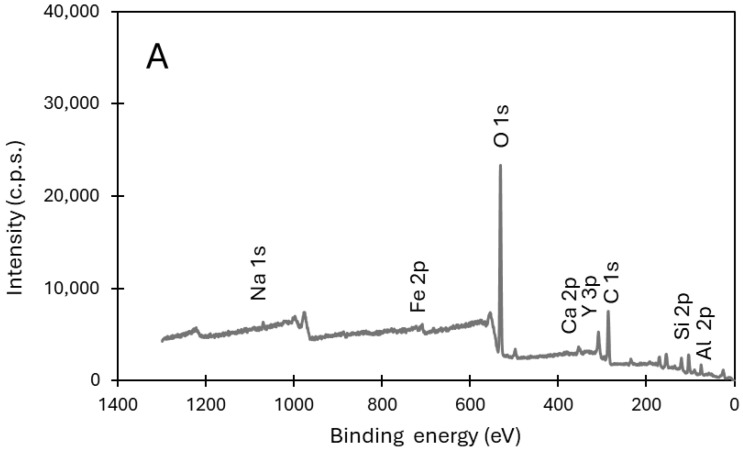

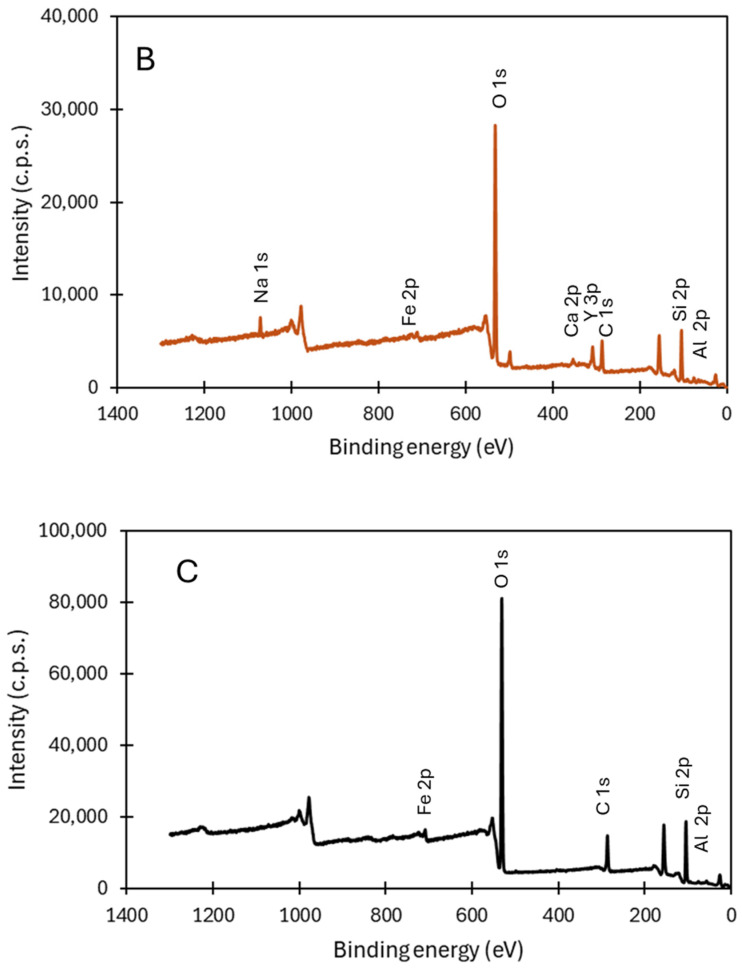
XPS survey spectrum of samples FA (**A**), FA_ZSM5_006_72 (**B**) and Fe/ZSM5_C (**C**).

ZSM-5 zeolite was synthesized from fly ash, using a two-step synthesis procedure, reported in the literature [30,34]. To optimize the synthesis, the amount of structuring agent tetrapropylammonium bromide (TPABr), added during the alkaline digestion step, and the duration of autoclave treatment were evaluated. For complete characterization, surface analysis by X-ray photoelectron spectroscopy (XPS) was performed on both the raw FA and on the zeolite sample FA_ZSM5_006_72—synthesized with a SiO_2_:TPABr ratio of 1:0.06 for 72 h in an autoclave at 160 °C (see detailed synthesis procedure below). A commercial ZSM-5 zeolite loaded with the same iron content as detected on FA, Fe/ZSM5_C, was also examined. The survey spectra are shown in Figure 1.

As expected, oxygen, silicon, carbon, iron, and aluminum were detected in all samples, including the iron introduced into the commercial ZSM-5. For FA and the derived zeolite sample FA_ZSM5_006_72, additional elements such as sodium, calcium and yttrium were also identified. It is noteworthy that these elements are present in both FA and FA-ZSM5_006_72 indicating that leaching during synthesis media is not significant and that the elements are incorporated into the zeolite structure. Table 1 presents the corresponding surface composition of FA, FA_ZSM5_006_72 and Fe/ZSM5_C samples.

As expected, oxygen, carbon, silicon and aluminum are the main elements. Sodium, iron and yttrium are present in substantially lower amounts. When comparing the surface composition of FA with that of the derived zeolite sample, FA_ZSM5_006_72, a decrease in carbon content is observed, attributable to the calcination step, and an increase in silicon due to the addition of SiO_2_ during zeolite synthesis. As mentioned before, the contents of sodium, iron and yttrium transferred from FA to FA_ZSM5_006_72 zeolite, are particularly relevant in the case of iron: the surface amount present on Fe/ZSM5_C is identical, suggesting that further addition of iron to the FA derived sample may not be required. This is especially important because the intended application of the produced zeolites is in Fenton reactions, where iron species serve as active sites. Nevertheless, the presence of other elements, namely yttrium, should not be disregarded, given the known influence of rare earth elements (REEs) on the electronic properties of surrounding elements.

**Figure 2:**
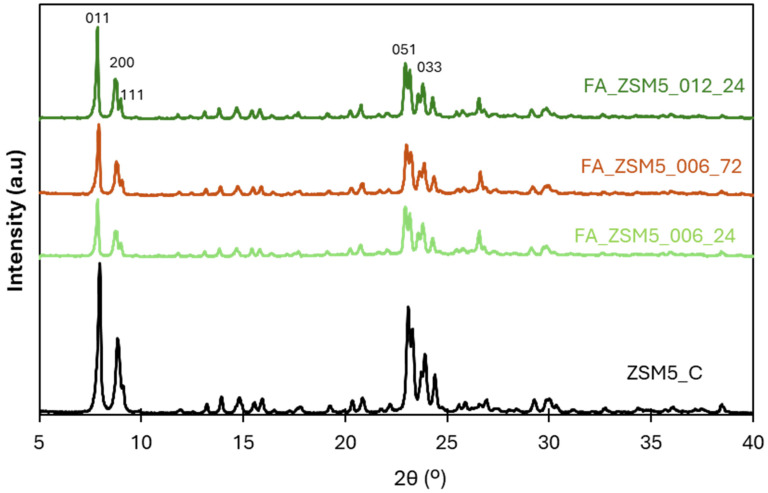
X-ray diffraction patterns of FA_ZSM5 samples and commercial ZSM-5. Miller indexes of the main diffraction peaks are indicated according to the IZA Database of Zeolite Structures (http://www.iza-structure.org/databases/ (accessed on 18 December 2025)).

Figure 2 shows the X-ray diffraction patterns of FA_ZSM5 zeolite samples, along with commercial ZSM-5 zeolite used as reference material.

The diffraction pattern of the starting FA material presents almost no crystalline phases, with only a few low intensity peaks ascribed to silica (quartz), mullite and magnetite [33]. On the other hand, FA-ZSM5 samples preset high intensity peaks typical of a crystalline material. When comparing all FA_ZSM5 samples with the commercial zeolite, ZSM5_C the diffraction patterns are essentially identical. To quantify the crystallinity percentage of the synthesized samples the procedure reported in ASTM D 5758-01 [35] was followed. The crystallinity percentage was obtained by dividing the cumulative area of the peaks between 22.44 and 25.15 ^0^2θ of each sample by the area of the correspondent peaks of commercial ZSM-5_C, using Peak-fit 4.12 software (Grafiti Inc., Palo Alto, CA, USA). The results obtained are presented in Table 2. As can be observed, all FA_ZSM5 samples present a crystallinity percentage of almost 80%. The first conclusion is that the variation in the operating conditions, namely the amount of surfactant added and the duration of the hydrothermal treatment, do not allow the samples to be differentiated. On the other hand, the lower crystallinity can also be attributed to the presence of structural defects, because of the synthesis conditions and thermal treatments during calcination procedure generating extra framework aluminum species (EFAl) [36].

**Figure 3:**
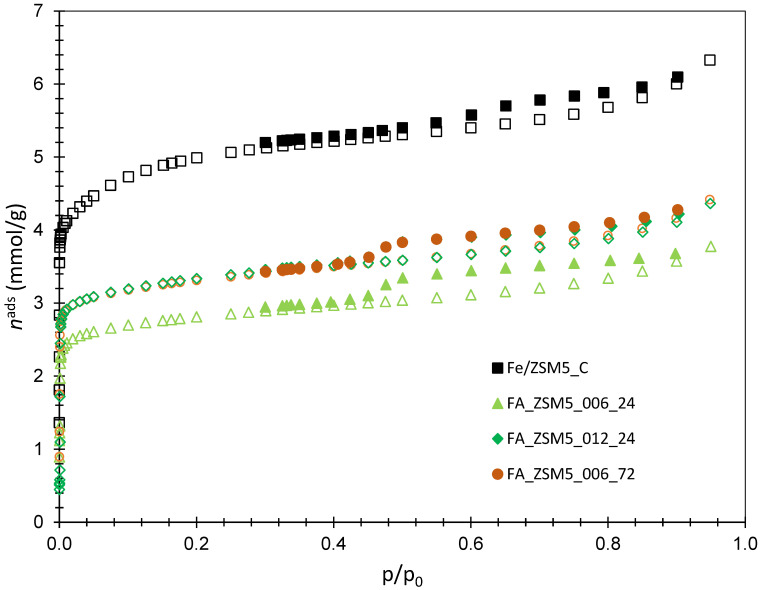
N_2_ adsorption–desorption isotherms at −196 °C for FA-ZSM5 samples and commercial ZSM5_C. The open and closed symbols represent adsorption and desorption, respectively.

The N_2_ adsorption–desorption isotherms at −196 °C for FA_ZSM5 samples and reference ZSM5_C are showed in Figure 3. The isotherm and respective textural parameters of FA were not included because of the material’s very low porosity; however, a previous study applied the BET method to estimate a specific surface area of 13.2 m^2^g^−1^ [33].

All the isotherms displayed in Figure 3 can be classified as a combination of type I + IV [37], showing, as expected, the prevalence of microporosity and some mesoporosity, denoted by hysteresis at relative pressure between 0.5 and 0.95. This hysteresis is attributed to particle aggregation. All zeolite samples prepared from FA display lower microporosity development compared with the commercial material ZSM5_C, since, in all cases, a notorious downward deviation in the low relative pressure region is noted. The textural parameters for all samples are depicted in Table 2, where microporosity was quantified using the α_s_ method, and a non-porous silica as reference material [38]. This method allows to distinguish the volume of ultramicropores (V_ulta_, ϕ < 0.7 nm) and supermicropores (V_super_, 0.7 nm < ϕ < 2 nm). The volume of the narrow pores, V_ultra_, is obtained from the line defined by experimental points at *p*/*p*^0^ > 0.02 up to, usually, *p*/*p*^0^ = 0.4, which corresponds to α_s_ = 1. The back extrapolation of the region defined by the points obtained at *p*/*p*^0^ > 0.4 corresponds to the total micropore volume, V_micro_, allowing to calculate the volume of the larger micropores, V_super_, through the difference between V_micro_ and V_ultra_. Mesopore volume, V_meso_, is calculated as the difference between the total pore volume, V_total_, and V_micro_, where V_total_ corresponds to the total amount of N_2_ uptake at *p*/*p*^0^ > 0.95, in line with the Gurvich rule [38].

In line with previous observations, it can be observed that for all FA-based samples the textural parameters are always lower when compared with the reference ZSM5_C. In general, there are no significant differences between FA_ZSM5 samples; however, a closer look reveals that V_ultra_ is slightly higher in the case of FA_ZSM5_012_24 and FA_ZSM5_006_72 samples. This suggests that the greater amount of TPABr added during synthesis or a longer hydrothermal treatment slightly impact the development of the narrow pores characteristic of the zeolitic framework. Conversely, compared with the commercial sample, V_super_ is substantially lower in all cases, whereas V_meso_, that results mainly from crystallites aggregation, presents slightly lower values for all FA_ZSM5 samples.

To evaluate the electronic properties of the samples, the measurements of magnetic susceptibility were performed in a balance, using the configuration of the traditional Gouy method. The mass susceptibility $\chi_{g}$ (in c.g.s.) units can be quantified using the general expression:

(5) $$\chi_{g}=\frac{l}{m}\left[C(R-R_{o})\right]+\chi_{\nu ,\;air}\;A$$

where *C* is the constant of proportionality of the balance obtained by calibration with a standard substance (HgCo(SCN)_4_), *R* is the reading obtained with the tube containing the sample, *R*_0_ is the reading of the empty tube, l is the sample length (cm) m is the sample weight (g), *A* is the cross-sectional area of the tube and *X*_*v*,*air*_ is the volume susceptibility of the displaced air. For powder samples, which is the present case, the term *X*_*v*,*air*_ *A* may be ignored. Table 3 presents the values of magnetic susceptibilities for all samples.

As expected, all samples present ferromagnetic behavior due to the presence of iron in their composition. However, all FA_ZSM5 samples present higher $\chi_{g}$ when comparing the solely iron-loaded Fe/ZSM5_C. Fly ash naturally contains pre-existing ferromagnetic phases that are retained during synthesis, like Magnetite phase (Fe_3_O_4_) that contributes significantly to the overall magnetic susceptibility of FA-ZSM5 samples, whereas the Fe-impregnated ZSM5_C sample lack this inherent contribution. Additionally, the higher $\chi_{g}$ may be attributed to interactions between iron and other elements present on FA. In fact, the influence of REE, particularly Y^3+^ on the magnetic properties of iron-containing materials, was reported in the literature [39]. The presence of REE on metal-loaded zeolites has been shown to reduce metal sintering while enhancing stability and dispersion, thereby positively affecting hydrogenation activity [40]. To our knowledge, the effect of yttrium inclusion on heterogenous Fenton catalysts has not yet been reported. However, Munoz et al. [41] reviewed several studies in which other elements, such as Mn or Co improved the oxidation rate and mineralization of magnetite-based catalysts by accelerating decomposition of H_2_O_2_ into ^●^OH radicals. Thus, it is reasonable to admit that the presence of yttrium (or traces of other elements, not detected on XPS survey) on FA_ZSM5 samples may exert a similar effect.

### 2.2. Catalytic Tests and Kinetic Studies

**Figure 4:**
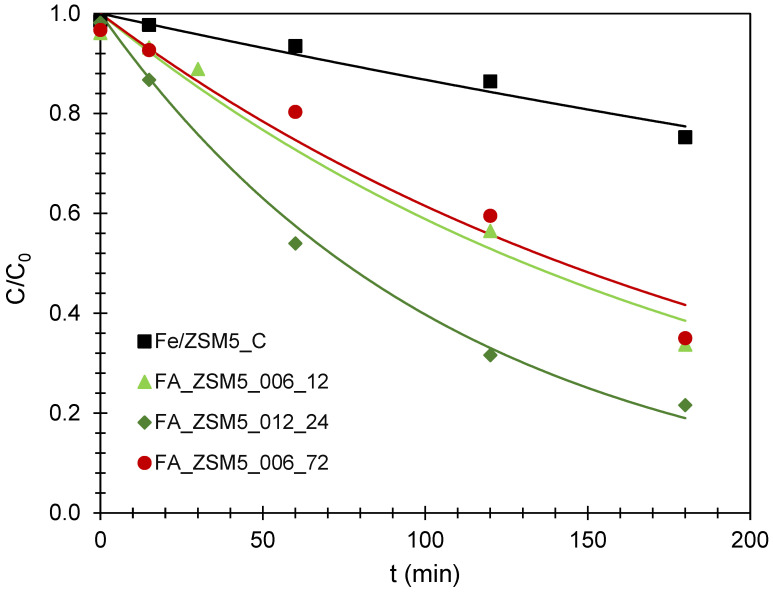
PA removal kinetic curves obtained from the application of pseudo-first-order kinetic model. The points representing the experimental data and respective coefficient of determination (*R*^2^) are included.

The performance of FA_ZSM-5 zeolites was evaluated in the removal of paracetamol (PA) via a Fenton reaction at 40 °C in the presence of H_2_O_2_. A commercial ZSM-5, only with H_2_O_2_, served as a control test; negligible PA removal was observed, leading to the conclusion that, in the chosen operating conditions, the reaction is effective only when Fe species are present. Figure 4 presents the kinetic curves, expressed as C/C_0_ vs. *t* where C is the PA concentration at a given reaction time and C_0_ is the initial PA concentration. After 1 h of contact between the heterogeneous catalysts and PA molecules, adsorption equilibrium was reached. The amount of PA adsorbed during the equilibration period was quantified. For all samples, the adsorption capacity values were below 3% (relative to the initial concentration of PA), indicating that the interaction occurs mainly at the catalyst external surface.

A first inspection of the kinetic profiles in Figure 4 reveals that all FA_ZSM5 samples show a more pronounced decay of C/C_0_ with time, when compared with the Fe/ZSM5_C reference sample. Despite these differences, a more rigorous analysis requires quantification through kinetic modeling.

The kinetic behavior was studied using pseudo-first-order and pseudo-second-order kinetic models, assuming that, regardless of the large number of reaction steps, Fenton reactions are described, albeit approximately, by one of these two kinetic models. The pseudo-first-order reaction law assumes that the rate of the reaction depends only on the concentration of one reagent, expressed as:

(6) $$\frac{\left[C\right]}{\left[C_{0}\right]}=e^{-k_{p1}t}$$

where [*C*] is the dye concentration, [*C*_0_] the initial dye concentration, *k*_p1_ is the pseudo-first-order kinetic constant and *t* is the reaction time. On the other hand, the pseudo-second-order rate law commonly assumes that the reaction rate depends on both reagent and surface sites [42] and can be expressed as:

(7) $$\frac{\left[C\right]}{\left[C_{0}\right]}=\frac{1}{k_{p2}[C_{0}]t+1}$$

being *k*_p2_ the pseudo-second-order kinetic constant and the other symbols keep the same meaning as stated before.

The application of pseudo-first-order and pseudo-second-order kinetic models in this study is justified by their widespread use in Fenton chemistry and their ability to capture the dominant mechanistic pathways—namely, the initial dependence on pollutant concentration and the interaction between hydroxyl radicals and the target contaminant. Although these simplified models neglect secondary effects such as mass-transfer limitations or radical quenching, they provide a tractable framework for interpreting the early-stage degradation kinetics observed in our experiments. It is important to acknowledge that Fenton reactions are intrinsically multi-step processes involving iron cycling, radical generation and recombination, and potential side reactions. The pseudo-kinetic approach therefore represents an approximation that prioritizes the most influential factors while remaining consistent with the experimental data [14,43].

The values of the rate constants of pseudo-first- and second-order are presented in Table 4. To validate the quality of the fit of non-linear equations to the experimental points, the following statistical parameters are also present: coefficient of determination (*R*^2^), the fit standard error of the regression (*S_Fit_*), and the Fisher–Snedecor parameter (*F*). In all cases, the number of experimental points used in the non-linear regression was five.

A brief inspection of the results shows that, under the chosen experimental conditions, the pseudo-first-order kinetic model is the one that best fits the experimental data, with the corresponding kinetic constant, *k*_p1_, higher when compared with the pseudo-second-order rate constant, *k*_p2_. These observations are in line with the literature for other contaminants [43,44] and with one of the few studies on paracetamol removal using heterogenous catalysts [45]. The fit to the pseudo-first-order model indicates that only the initial phase of the degradation process is occurring since, as can be visualized in Figure 4, a true plateau was never reached even after 180 min of reaction.

In line with the kinetic profiles in Figure 4, the values obtained for rate constants depicted on Table 4 show substantially higher values for all FA_ZSM5 samples when compared with Fe/ZSM5_C. These results cannot be explained by the classic crystallinity and texture parameters (Table 2) since in all cases FA_ZSM5 samples present lower crystallinity and V_micro_ than the reference catalyst. To rationalize these results it must be noted that when operating at a pH of around 4, as in the case of this work, PA molecules may exist as dimers [46,47]. Their estimated dimensions (in nm) 1.58 (length) × 1.19 (width) × 0.46 (thickness), are incompatible with the elliptical pore openings of ZSM-5 zeolite (in nm) 0.51 × 0.55 and 0.53 × 0.56 [48]. Accordingly, most Fenton reactions likely occur at the external surface of zeolite crystals or pore openings.

This assumption justifies the low rate constant and PA removal after 180 min observed in the assay with Fe/ZSM5_C, but not the higher values observed with FA_ZSM5 samples. The structural and textural parameters of FA_ZSM5 samples do not present enough variability to justify the differences between them. However, they all have a disordered fraction (about 20% crystallinity lower than the reference material), which can reduce diffusional constraints and improve catalytic performance relative to Fe/ZSM5_C. In fact, structural defects and consequent EFAl species can have a beneficial role on the catalytic performance. A classic example is the improved catalytic behavior of FAU zeolite in fluid catalytic cracking (FCC) where EFAl species are generated through steaming [49] or, more recently, the introduction of aluminon cations followed by calcination, leading to an increase in EFAl species, with a positive impact on the transformation 4-tert butyl cyclohexanone with isopropanol in the presence of Y-modified zeolite [50].

XPS analysis of a representative FA_ZSM5 sample shows an identical surface iron content, but also detects yttrium, which may affect catalytic performance. Other authors have studied the effect of yttrium inclusion in heterogeneous catalysts. For instance, Wang et al. [51] studied the role of Y inclusion on Ce/Ni-Metakaolin and its effect on the catalytic performance in CO_2_ methanation. The authors showed that the presence of yttrium promotes the creation of oxygen vacancies which increases the nickel dispersion, resulting in increased activity on CO_2_ methanation. On the other hand, Zhang et al. [52] and Li et al. [53] studied the prominent role of oxygen vacancies in the formation of hydroxyl radicals to promote Fenton reaction by weakening the O-O bond of adsorbed H_2_O_2_, which benefits the electron transfer by reducing its decomposition activation energy. As a result, a higher amount of ^●^OH species is produced, improving the efficiency in Fenton reaction. In this study we assume that the results can be interpreted considering previous literature showing increased magnetic susceptibility of FA-ZSM5 materials—especially for sample FA_ZSM5_012_24. 

These combined effects positively influence catalytic performance, *k*_p1_ is about 1.6–1.8 times higher than for FA_ZSM5_006_24 and FA_ZSM5_006_72, respectively, and more than 6 times higher than for Fe/ZSM5_C.

### 2.3. pH Effect on PA Degradation

**Figure 5:**
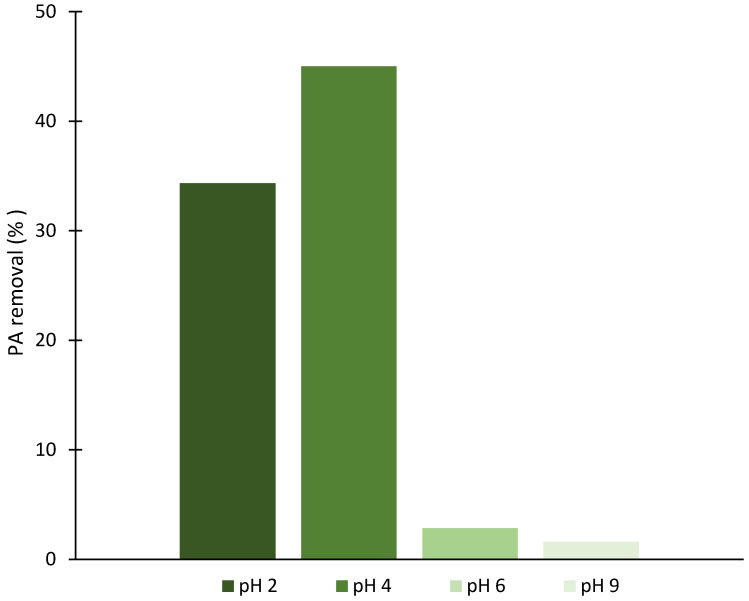
Removal percentage of PA after 1 h reaction at 40 °C, changing pH from 2 to 9 for FA_ZSM5_012_24 sample. Experimental conditions: 10 mg catalyst, 25 mL of 20 ppm PA solution, stirring at 400 rpm, H_2_O_2_ (70 mM, 0.5 mL) added to initiate reaction.

To investigate the effect of pH on PA degradation, a series of catalytic assays was performed using the FA_ZSM5_012_24 sample; while keeping all other experimental parameters constant, the reaction was carried out at pH values from 2 to 9. Figure 5 presents the PA degradation percentage at each pH value after 1 h of reaction.

As shown in Figure 5, PA removal efficiency remains high at low pH values (pH 2 and 4) but exhibits a drastic decrease at pH 6 and 9. These results align with established literature on heterogeneous Fenton reactions, where substantial degradation efficiency loss is observed at pH > 4 [54,55]. This behavior can be ascribed to the presence of excess H_2_O_2_ that readily decomposes into H_2_O and O_2_ under alkaline conditions, thereby reducing ^●^OH radical production. Consequently, optimal Fenton activity occurs in acidic conditions (pH 2–4), while alkaline environments promote non-productive H_2_O_2_ decomposition rather than ^●^OH radical generation, resulting in significantly slower degradation reactions [56,57]. The lower PA removal at pH 2 can be attributed to the formation of stable coordination complexes and excessive leaching of iron ions from the solid catalyst into the solution [58,59].

### 2.4. Regeneration Studies

**Figure 6:**
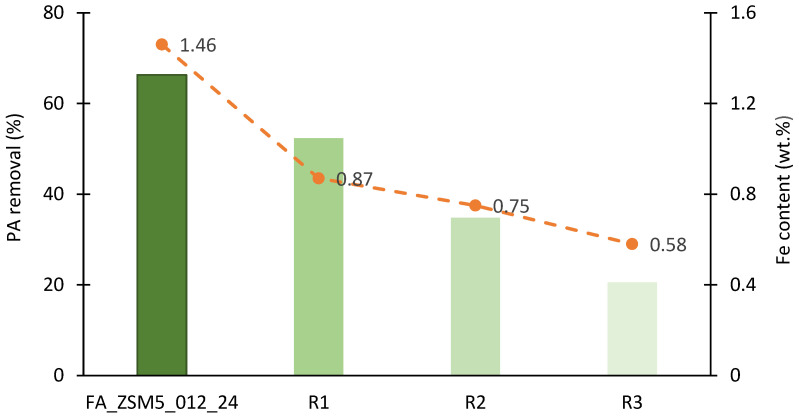
PA removal percentage (green bars) and Fe content (orange dashed line) for the FA_ZSM5_012_24 sample and three consecutive reuse cycles (R1–R3). Reaction at 40 °C, pH = 4 for 2 h with thermal regeneration between each cycle (350 °C, 4 h). Experimental conditions: 10 mg catalyst, 25 mL of 20 ppm PA solution, stirring at 400 rpm, and H_2_O_2_ (70 mM, 0.5 mL) added to initiate the reaction.

To investigate the ability to reuse the FA-based catalysts, FA_ZSM5_012_24 sample—previously identified as the best performing catalyst—was submitted to four consecutive catalytic runs under identical conditions. Thermal regeneration between each cycle was carried out at 350 °C for 4 h, with a heating rate of 5 °C min^−1^. The results presented in Figure 6 show the PA removal percentage after 2 h of reaction.

As can be seen, there is a progressive decrease in PA removal with the number of reuse cycles. There is an approximate 20% drop from the fresh sample to the first reuse cycle (R1), followed by more pronounced decreases of almost 50% and 70% for R2 and R3, respectively, when compared to the fresh catalyst. This decline correlates clearly with the progressive decrease in Fe content within the zeolite structure due to leaching phenomena, as depicted by the orange dashed line in Figure 6.

**Figure 7:**
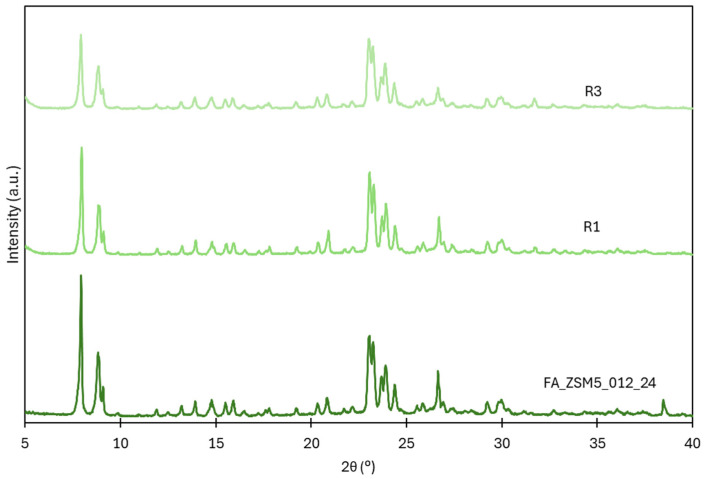
X-ray diffraction patterns of FA_ZSM5_012_24 sample and first (R1) and third (R3) reuse cycles.

No significant loss of crystallinity during the reuse cycles was observed, as seen in Figure 7.

An investigation on lixiviation effects was carried out on fresh samples FA_ZSM5_006_72 and FA_ZSM5_012_24 as well as the reference Fe/ZSM5_C catalyst. After a 1 h equilibration time, the solid was removed by centrifugation and the reaction was conducted in the usual manner. After 45 min the reaction was stopped, and PA absorbance was measured. In all cases, the ratio between PA concentration after 45 min reaction and the initial concentration was C/C_0_ ≈ 1, indicating that the reaction did not occur in homogeneous media. This may be explained considering the released species were present in insufficient amounts to catalyze PA degradation in detectable amounts after 45 min. Additionally, deposition of reaction products and sub-products on the zeolite surface, ineffectively removed during thermal regeneration, should also be considered. This explanation is strengthened by the observation that after R3 there was a ~30% decrease in the total porous volume of the zeolite samples while maintaining the crystalline structure typical of ZSM-5 material, denoting that the chosen regeneration conditions were insufficient to remove all organic matter from the heterogeneous catalyst.

## 3. Materials and Methods

### 3.1. Catalysts Preparation

The starting fly ash (FA) material originated from a Portuguese thermoelectric power plant utilizing Colombian bituminous coal for combustion. Fly ash is captured via electrostatic precipitators and subsequently stored in silos, where our sample was collected. Upon analysis, the FA sample was classified as non-compliant for sale, and is therefore considered waste. In this study the FA sample was quartered and used without further treatment. The chemicals used for the zeolite synthesis and catalytic experiments were purchased from Merck (Darmstadt, Germany), and used as received.

The synthesis of ZSM-5 zeolite (MFI structure) followed published protocols [30,34], with some adaptations. Briefly, 1.5 g of FA was suspended in a 2 M NaOH solution at 90 °C, on a heating plate with temperature control (IKA C-MaHS7, Staufen, Germany). Then, 3.413 g of SiO_2_ were added to adjust the global Si/Al ratio to 12 and the required amount of tetrapropylammonium bromide (TPABr) to attain the molar ratio 1 SiO_2_: 0.06 or 0.12 TPABr. After 3 h mixing at 210 rpm the suspension was transferred to stainless steel PTFE-lined autoclaves and heated at 160 °C under autogenous pressure for 24 or 72 h. The material was recovered by filtration, washed until pH around 7, dried overnight and calcined at 540 °C for 5 h, with a heating ramp of 5^0^ min^−1^ (Nabertherm B170, Bahnhofstr, Germany). The samples were designated as: FA_ZSM5_x_y, where x is the TPABr added (molar ratio) and y corresponds to the time spent inside the autoclave (h). A commercial ZSM-5 zeolite (Zeolyst, Conshohocken, PA, USA), with a SiO_2_/Al_2_O_3_ = 30 (CBV3024E, Lot2200-99) was used as a reference material. About 4 wt.% of iron was introduced by incipient wetness impregnation method, using the amount of water correspondent to the previously determined porous volume of the solid. This amount of water was used to dissolve Fe(NO_3_)_3_. 9H_2_O (>99%) and mix with the solid until a paste was formed. The sample was dried and calcined at 350 °C, with a heating rate of 5^0^ min^−1^.

### 3.2. Physicochemical Characterization

The samples were analyzed by inductively coupled plasma–optical emission spectroscopy ICP-AES at Laboratório de Análises, IST, Lisbon, to probe the chemical composition of FA. Surface composition was studied by X-ray photoelectron spectroscopy, XPS, performed at INCAR, Oviedo. The structural characterization of parent FA and produced zeolites was assessed from X-ray powder diffraction (XRD) patterns, acquired at room temperature using a Pan’Analytical PW3050/60X’Pert PRO (θ/2θ) diffractometer (Phillips, Almelo, The Netherlands), equipped with X’Celerator detector with automatic data acquisition (X’Pert Data Collector (v2.0b) software), using a monochromatized CuKα radiation as incident beam, 40 kV-30 mA. The scans were made in a 2θ range of 5–40° with a step size of 0.017° 2θ and a time per step of 0.6 s. N_2_ adsorption/desorption isotherms were performed to characterize the textural properties of the samples. The assays were made in an automatic apparatus ASAP2010 (Micromerics Instruments Corporation, Norcross, GA, USA). Prior to each experiment the samples (about 50 mg) were outgassed at 300 °C for 3 h under vacuum greater than 10^−2^ Pa. The magnetic susceptibility measurements were performed in a balance MSK-MK1 (Sherwood Scientific, Cambridge, UK).

### 3.3. Catalytic Tests

The catalytic tests were performed following a previously optimized procedure [14]. In brief, 10 mg of the samples was accurately weighted and placed in 50 mL Falcon type tubes. Then, 25 mL of 20 ppm paracetamol (PA) solution was added to each tube and the tubes were immersed in a thermostatic bath at 40 °C (Julabo MP, Seelbach, Germany). The bath was positioned on a multiposition magnetic stirrer (Selecta Multimatic 9-S, Barcelona, Spain), with the stirring speed set to 400 rpm. The system was left to reach adsorption equilibrium for 1 h. This equilibrium stage was optimized previously [14] and the reaction was initiated only after it was achieved. Immediately before starting the reaction, the pH was adjusted to values of 2, 4, 6 and 9 (using a 0.5 M HCl solution) to investigate the effect o pH; for all other assays the pH was set to 4. The reaction began by adding 0.5 mL of a 70 mM H_2_O_2_ solution to each tube. At predetermined time points, the reaction was stopped by adding 3 drops of 0.5 M NaOH solution. The solid was separated from the reaction medium by centrifugation (Hermle, Z206A, Gosheim, Germany), with a recovery rate of 80–85%. The absorbance of the remaining solution was measured with a double-beam spectrophotometer (VWR, UC-6300PC, Radnor, PA, USA) using a 10 mm optical length quartz cell and water as reference. For each sample at every specified time point, three independent aliquots were collected from a single Falcon tube and analyzed separately. This approach ensured a standard deviation below 5%. Before the measuremets, a calibration curve was prepared with PA solutions at concentrations that produced absorbances ranging from 0.15 and 1.0, thereby satisfying the Beer–Lambert law.

## 4. Conclusions

ZSM-5 zeolite was successfully synthesized from fly ash waste with SiO_2_ supplementation to match commercial material composition, by optimizing both structure-directing agent concentration and hydrothermal treatment duration. The obtained materials displayed diffraction patterns characteristic of MFI zeolite structure, with low impurity content, while the N_2_ isotherm showed a profile typical of zeolites. Although the textural parameters were lower than those of commercial ZSM-5 zeolite. Beyond the expected Si and Al contents, significant amounts of Fe and Y were detected in the FA and incorporated into the synthesized samples. FA-ZSM5 samples showed increased magnetic susceptibility compared with iron-only loaded commercial ZSM-5, indicating synergistic effects between iron and other elements such as yttrium that migrate from FA to the derived zeolite samples. This effect was most pronounced for the sample with a high amount of structuring agent (FA_ZSM5_012_24), suggesting that the quantity of structuring agent influences metal ion distribution or reflects the heterogeneity of the raw FA. Catalytic performance in the removal of paracetamol via Fenton reaction showed enhanced PA removal by FA_ZSM5 samples compared with Fe/ZSM5_C. Two main interpretations may explain this behavior: (i) Surface site catalysis—the catalytic reactions likely occur at the external surface of the zeolite crystals, as PA molecules tend to form dimers that cannot readily diffuse into the zeolite pore structure. A more disordered FA_ZSM5 structure can therefore benefit the reaction by providing additional active sites; (ii) Yttrium-enhanced radical formation—yttrium may promote the generation of ^●^OH radicals, through oxygen vacancies creation, especially in FA_ZSM5_012_24, further boosting catalytic activity. Regeneration and reuse studies showed a progressive decrease in PA removal efficiency, with a significant drop of almost 70% relative to the fresh catalysts after repeated cycles due to Fe leaching.

Overall, these preliminary results demonstrate that fly ash—classified as non-compliant for sale—can be valorized into a zeolite-based Fenton catalyst. The rich Fe content of FA eliminates the need for additional activation steps, and the presence of other elements (e.g., Y) appears to enhance catalytic performance. Encouraged by these findings, further kinetic, mechanistic studies and environmental impact (toxicity) will be pursued, along with investigations into the removal of other contaminants such as industrial organic dyes.

## Figures and Tables

**Table 1:** Surface contents determined from XPS atomic percentages for FA, Fa_ZSM5_006_72, and Fe/ZSM5_C samples.

Element (at%)	FA	FA_ZSM5_006_72	Fe_ZSM5_C
O	40.72	45.49	47.66
C	31.72	16.80	18.66
Si	12.82	28.61	31.60
Al	9.05	4.61	1.41
Na	0.63	1.27	-
Fe	0.43	0.68	0.68
Y	2.81	1.73	-

**Table 2:** Degree of crystallinity and textural parameters obtained from N_2_ adsorption isotherms for FA_ZSM5 samples, commercial ZSM-5 before (ZSM-5) and after iron loading (Fe/ZSM5_C).

Sample	C_XRD_^1^ (%)	V_super_ (cm^3^ g^−1^)	V_ultra_ (cm^3^ g^−1^)	V_micro_^2^ (cm^3^ g^−1^)	V_meso_^3^ (cm^3^ g^−1^)	A_ext_ (m^2^ g^−1^)
ZSM-5_C	100	0.05	0.10	0.15	0.07	35
FA_ZSM5_006_24	78	0.01	0.07	0.08	0.05	34
FA_ZSM5_012_24	79	0.02	0.08	0.10	0.05	35
FA_ZSM5_006_72	78	0.02	0.08	0.09	0.06	35

^1^ Degree of crystallinity (C_XRD_) calculated from powder X-ray diffraction patterns, using commercial ZSM-5 as reference. ^2^ Microporous volume, V_micro_, and external surface area A_ext_, quantified through the application of α_s_ method; ^3^ Mesoporous volume, V_meso_ = V_total_ − V_micro,_ where the total volume (V_total_) corresponds to the amount of N_2_ adsorbed at *p*/*p*^0^ ≈ 0.95.

**Table 3:** Magnetic susceptibility ($\chi_{g}$) measurements for FA_ZSM5 samples and reference ZSM- Fe/ZSM5_C at 25 °C.

Sample	$\chi_{g}$ (c.g.s.) × 10^5^
Fe/ZSM5_C	1.4
FA_ZSM5_006_24	5.1
FA_ZSM5_012_24	9.2
FA_ZSM5_006_72	2.9

**Table 4:** Rate constants of pseudo-first-order (*k*_p1_) and pseudo-second-order (*k*_p2_) for the kinetic curves of PA removal, correspondent statistical parameters *R*^2^, *S_Fit_* and *F* (see description in the text).

**Sample**	**Pseudo-First-Order Kinetic Model**			
	***k*_p1_ (min^−1^) × 10^−3^**	***R* ^2^**	***s*_Fit_**	***F***
Fe/ZSM5_C	1.4 ± 0.2	0.962	0.395	76.4
FA_ZSM5_006_24	5.3 ± 0.6	0.979	0.970	137.0
FA_ZSM5_012_24	9.2 ± 0.4	0.996	0.530	787.6
FA_ZSM5_006_72	4.9 ± 0.7	0.962	1.179	75.4
**Sample**	**Pseudo-Second-Order Kinetic Model**			
	***k*_p2_ (ppm^−1^ min^−1^) × 10^−4^**	***R* ^2^**	***s*_Fit_**	***F***
Fe/ZSM5_C	0.9 ± 0.1	0.949	0.459	55.7
FA_ZSM5_006_24	3.6 ± 0.8	0.944	1.562	51.0
FA_ZSM5_012_24	7.2 ± 0.9	0.984	1.077	188.3
FA_ZSM5_006_72	3.3 ± 0.8	0.918	1.728	33.5

## Data Availability

Data are contained within the manuscript.
